# Left Ventricular Non-Compaction Cardiomyopathy: A Review of the Pathophysiology, Epidemiology, Diagnosis, Genetics, and Clinical Management

**DOI:** 10.3390/jpm15100484

**Published:** 2025-10-10

**Authors:** Luis Elias Martínez-Tittonel, Florin Radu Ciorba, Xavier Bayona-Huguet, Edgardo Kaplinsky

**Affiliations:** 1 Department of Primary Care, Badalona Serveis Assistencials, Hospital Municipal de Badalona, 08911 Badalona, Spain; fcciorba@bsa.cat; 2 Department of Research, Innovation, and Clinical-Management Projects, Hospital Municipal de Badalona, 08911 Badalona, Spain; xbayona@bsa.cat; 3 Cardiology Unit, Badalona Serveis Assistencials, Hospital Municipal de Badalona, 08911 Badalona, Spain; ekaplinsky@bsa.cat

**Keywords:** left ventricular non-compaction, LVNC, genetics, embryogenesis, notch, BMP10, cardiac magnetic resonance, LGE, T1/ECV, fractal, precision medicine

## Abstract

Left ventricular non-compaction cardiomyopathy (LVNC) is an uncommon myocardial phenotype characterized by prominent trabeculae and deep blood-filled recesses. The expanding use of cardiac magnetic resonance (CMR) has increased detection, yet uncertainty persists about whether LVNC is a distinct disease or a phenotype that overlaps with other cardiomyopathies. LVNC expression reflects the interplay among genotype, sex, ancestry, and hemodynamic load and thus serves as a model for precision cardiology. We conducted a narrative review of literature published between January 2000 and April 2025 in major databases. We included clinical studies with at least 10 patients, meta-analyses, reviews, and consensus statements addressing pathophysiology, genetics, diagnosis, prognosis, and treatment. Sarcomeric variants account for a substantial fraction of cases and connect LVNC with dilated and hypertrophic cardiomyopathies. Echocardiographic and CMR criteria identify the phenotype but blur the boundary between physiological and pathological hypertrabeculation. Fibrosis on late gadolinium enhancement and systolic dysfunction are consistently associated with worse outcomes. Current management largely adapts heart-failure strategies, including neurohormonal blockade, SGLT2 inhibitors, and implantable cardioverter-defibrillators in selected high-risk patients. Optimal care integrates clinical, imaging, and genetic information. The lack of universal diagnostic criteria highlights the need for prospective studies and consensus to standardize diagnosis and treatment. Future algorithms that combine multi-omics, quantitative imaging, and AI-based risk prediction could individualize surveillance, pharmacotherapy, and device therapy.

## 1. Introduction

Left ventricular non-compaction cardiomyopathy (LVNC) is characterized by a spongy ventricular architecture with thick trabeculae and deep blood-filled recesses [1]. It was initially attributed to embryonic compaction failure between gestational weeks five and eight; however, contemporary genetic evidence places LVNC within a spectrum shared with dilated and hypertrophic cardiomyopathies [2,3,4,5]. The European Society of Cardiology lists LVNC among “unclassified” cardiomyopathies, whereas the American Heart Association includes it among primary congenital forms [3]. Apparent prevalence varies with imaging technique and population: Ross et al. reported 1.05% in healthy controls, 3.16% in athletes, and up to 18.6% in pregnant women using echocardiography [6]. Cardiac magnetic resonance increases detection to 14.8%, and the disparity between criteria illustrates a considerable risk of over-diagnosis [7,8]. Described phenotypes include isolated non-compaction with preserved function; dilated and hypertrophic variants that overlap with DCM and HCM [2,3,9]; forms associated with congenital heart disease (e.g., tetralogy of Fallot) [3]; and acquired, reversible hypertrabeculation observed in pregnancy, athletes, and sickle-cell anemia [7,10]. In the current era, cardiomyopathies are increasingly approached through a precision-medicine lens that tailors diagnostics and therapy to the individual’s molecular and phenotypic profile. LVNC—with marked genotype–phenotype variability and reversible forms (e.g., pregnancy, athletic remodeling)—offers a unique opportunity to apply and test such approaches [11,12]. As summarized in Table 1, apparent prevalence reflects the proportion meeting at least one echocardiographic or CMR morphological threshold and varies by cohort and modality.

Objective of the review: To comprehensively describe the pathophysiology, epidemiology, clinical presentation, diagnostic methods, differential diagnosis, treatment, and research perspectives of LVNC using evidence available up to April 2025.

## 2. Materials and Methods

We conducted a narrative review addressing the pathophysiology, epidemiology, genetics, clinical presentation, diagnosis, prognosis, and management of left ventricular non-compaction cardiomyopathy (LVNC). Searches were performed in PubMed, Google Scholar, and CrossRef from January 2000 to April 2025. No language restrictions were applied provided that an English abstract was available. We additionally screened the reference lists of included publications to identify relevant studies not captured by the electronic searches.

Eligibility criteria: We considered the following for inclusion: (i) human clinical studies enrolling ≥10 individuals with LVNC; (ii) genetic studies (family series, multigene panels, exome/genome) with phenotypic characterization of LVNC; (iii) imaging studies with diagnostic or prognostic implications; (iv) mechanistic, animal, or cellular models directly relevant to trabeculation/compaction pathways; and (v) narrative or systematic reviews, meta-analyses, position statements, or consensus documents. We excluded isolated case reports without mechanistic contribution, editorials or letters without original data, and series with ambiguous or mixed cardiomyopathic phenotypes when LVNC could not be distinguished.

Study selection and data extraction: Two reviewers independently screened titles/abstracts and assessed full texts for eligibility. From each eligible article we extracted study design, population, LVNC definition, imaging/genetic methods, and clinically relevant outcomes (heart failure events, arrhythmias, thromboembolism, transplantation, or mortality). When available, identifiers (DOI/PMID) were verified for accuracy. Disagreements were resolved by discussion and consensus.

Synthesis approach: Given the heterogeneity of definitions, imaging thresholds, and study designs, we synthesized the evidence qualitatively and structured it by domain (etiology/pathophysiology, clinical presentation, diagnosis, prognosis/risk stratification, treatment, and differential diagnosis). Quantitative pooling was not attempted because between-study variability in LVNC criteria and outcome definitions precluded a meaningful meta-analysis.

Administrative note: This is a narrative review that does not involve human participants or individual patient data beyond published reports; ethical approval and consent were therefore not required.

## 3. Etiology and Pathophysiology

LVNC arises from the interplay between genetic variants, developmental programs of trabeculation/compaction, and hemodynamic and mechanobiologic cues. Sarcomeric genes are most frequently implicated, particularly MYH7 and ACTN2, with TTN truncating variants contributing in a substantial subset; overlap with dilated and hypertrophic cardiomyopathies is common, supporting a continuum rather than a discrete entity in many families [2,3,5]. Additional contributors include nuclear-envelope genes (e.g., LMNA), ion-channel and conduction genes (e.g., HCN4, SCN5A), developmental transcription factors (NKX2-5, PRDM16, TBX20), and mitochondrial pathways, each modulating penetrance, age at presentation, and associated extracardiac features [2,3,5,9].

Developmentally, trabeculation precedes compaction; signaling axes such as NOTCH/BMP10 and neuregulin–ErbB orchestrate endocardial–myocardial crosstalk, while altered mechanotransduction and flow patterns can skew trabecular architecture [3,13]. In adults, loading conditions and mechanosensitive pathways (e.g., MAPK–AKT, Hippo/YAP–TAZ, and TGF-β remodeling) influence phenotype expression, fibrotic remodeling, and energetics, helping explain reversible hypertrabeculation in pregnancy or with intensive athletic training, as well as progression to ventricular dysfunction in genetically primed myocardium [3,13].

Imaging correlates of injury are integral to pathobiology. Late gadolinium enhancement (LGE) reflects extracellular matrix expansion and correlates with adverse outcomes across cohorts [2]. T1/T2 mapping and deformation indices (e.g., global longitudinal strain) capture diffuse disease and subclinical dysfunction that may precede overt changes in ejection fraction, refining risk classification when morphology alone is equivocal [12]. Genotype-specific clinical signals are increasingly recognized: LMNA variants track with early conduction disease and atrial/ventricular arrhythmias; HCN4 with sinus-node dysfunction and occasional aortic dilation; SCN5A with conduction disease and ventricular arrhythmias; and selected developmental genes with congenital phenotypes and pediatric-onset LVNC [2,5,9]. Familial aggregation and variable expressivity justify genetic counseling and cascade testing, with longitudinal follow-up tailored to genotype, injury markers, and clinical course. Key genotype–phenotype patterns and practical management signals are summarized in Table 2.

## 4. Clinical Presentation and Natural History

LVNC exhibits a broad clinical spectrum ranging from incidental imaging findings with preserved systolic function to overt heart failure with malignant arrhythmias. Common presentations include exertional dyspnea, fatigue, palpitations, presyncope/syncope, chest discomfort, and signs of pulmonary or systemic congestion when heart failure develops. Atrial fibrillation is frequent, and sustained ventricular tachycardia tends to cluster in patients with reduced LVEF and/or myocardial fibrosis. Thromboembolic risk increases in the presence of apical recesses, atrial fibrillation, reduced LVEF, or documented apical thrombus; anticoagulation should be individualized based on arrhythmia status, imaging evidence of thrombus, and overall risk profile [16,17].

Population-specific contexts deserve tailored interpretation. Pregnancy may unmask transient hypertrabeculation that fulfills morphological thresholds yet often regresses postpartum; reassessment 6–12 months after delivery is recommended before assigning a lifelong diagnosis. Competitive athletes frequently show increased trabeculation related to physiological remodeling with preserved LVEF and no LGE; when doubt persists, short-term de-training and multiparametric reassessment help avoid overdiagnosis. Pediatric LVNC frequently coexists with congenital heart disease or metabolic disorders, and outcomes depend on baseline function, arrhythmias, and syndromic features [14,18,19]. Familial aggregation is common; genetic counseling and cascade screening enable early identification of at-risk relatives. Across ages, natural history is shaped by the interaction between genotype, loading conditions, and tissue injury. In asymptomatic individuals with preserved function and no injury markers, a conservative strategy with periodic clinical and imaging follow-up is appropriate; escalation is guided by development of systolic dysfunction, fibrosis on LGE, clinically significant arrhythmias, or thromboembolic events [7,10,20]. Typical clinical scenarios, red flags and next actions are outlined in Table 3.

## 5. Diagnosis

Diagnostic assessment of LVNC should balance morphology with evidence of myocardial injury and the clinical context. In low pretest-probability settings (healthy adults, athletes, late pregnancy), morphology alone has limited positive predictive value; combining structural thresholds with injury markers improves specificity and clinical usefulness. Operational diagnostic thresholds and injury markers are consolidated in Table 4. Features that favor physiological hypertrabeculation in athletes and pregnancy are listed in Table 5.

Echocardiography: Three commonly used echocardiographic approaches include the Chin ratio (X/Y ≤ 0.5 in diastole) and the Jenni criterion (non-compacted to compacted ratio, NC/C > 2 in systole, with a bilayered myocardium and perfused intertrabecular recesses on color Doppler) [10,15]. Technical pitfalls include suboptimal apical windows, through-plane motion, and load dependence. When suspicion is high, targeted apical views, careful caliper placement at end-systole/end-diastole as specified by the criterion, and consideration of 3D echocardiography can reduce misclassification. Additional echocardiographic clues such as thinning of the compact layer and impaired deformation (reduced global longitudinal strain) support disease when present, but they are not diagnostic in isolation [27].

Cardiac magnetic resonance (CMR): CMR improves reproducibility and whole-heart coverage. The Petersen definition uses a diastolic NC/C ≥ 2.3, while the Jacquier approach quantifies trabeculated mass and considers values > 20% of LV mass consistent with LVNC in validation cohorts [7,8]. Segmentation method, reader experience, and vendor/software choices influence results and account for some variability across studies. Beyond morphology, late gadolinium enhancement (LGE) identifies fibrosis and correlates with adverse outcomes independent of LVEF; T1/T2 mapping and strain analysis (feature-tracking GLS) detect diffuse disease and may flag higher-risk phenotypes when ejection fraction is preserved [1].

ECG/CT/Biopsy: ECG frequently demonstrates non-specific conduction delay, bundle-branch block, or fragmented QRS; these findings variably correlate with fibrosis and arrhythmic risk. Cardiac CT can delineate trabeculation and apply NC/C thresholds in patients with MRI contraindications. Endomyocardial biopsy is reserved for suspected infiltrative/storage disease or myocarditis when results would alter management.

Diagnostic framing: A pragmatic approach is to require morphology plus at least one injury marker (fibrosis on LGE, reduced LVEF/GLS, or malignant arrhythmias/thromboembolism) before making a firm diagnosis in low pretest-probability scenarios. Familial aggregation, syndromic features, or high-risk genotypes (e.g., LMNA) increase pretest probability and may justify earlier risk stratification steps.

## 6. Prognosis and Risk Stratification

Prognosis in LVNC is driven less by morphology per se and more by markers of myocardial injury, electrical instability, and pump failure. Across cohorts, reduced LVEF, late gadolinium enhancement (LGE), and non-apical extension of trabeculation consistently associate with higher major adverse events (heart failure hospitalization, ventricular arrhythmias, stroke/systemic embolism, transplantation, or death) [2,8,21,22]. In asymptomatic individuals with preserved function, a conservative strategy is reasonable unless injury markers emerge. When genotype is known, risk stratification should integrate variant-specific signals; for example, LMNA variants carry earlier conduction disease and arrhythmic events and may justify a lower threshold for device therapy when combined with imaging or clinical risk features [13,15,22]. Tissue characterization with LGE and deformation imaging (reduced GLS) refine risk beyond LVEF and help identify phenotypes at higher arrhythmic or heart-failure risk despite apparently preserved ejection fraction [2,12]. In practice, individualized risk models that combine clinical variables (age, symptoms, AF), imaging (LVEF, LGE, GLS, non-apical extension), and genotype support decisions on surveillance intensity, ICD consideration, anticoagulation, and exercise restrictions [3,22,23,24,25]. Prognostic modifiers and a stepwise risk framework are summarized in Table 6.

## 7. Treatment

Management of LVNC largely adapts evidence from non-ischemic cardiomyopathy and heart failure, tailoring decisions to tissue injury, arrhythmic burden, thromboembolic risk, and genotype. In asymptomatic individuals with preserved systolic function and no injury markers (no LGE, normal GLS, no significant arrhythmias), a conservative strategy with periodic clinical review, ECG/ambulatory monitoring, and repeat imaging is appropriate. Once heart failure symptoms or systolic dysfunction develop, initiate guideline-directed medical therapy (GDMT) with an ACE inhibitor or ARB or sacubitril–valsartan, a beta-blocker, a mineralocorticoid receptor antagonist, and an SGLT2 inhibitor, titrating to guideline targets as tolerated [28]. After ≥3 months of optimized therapy, patients with LVEF ≤ 35% should be considered for primary-prevention ICD following major society recommendations for non-ischemic cardiomyopathy; in those with left bundle branch block and QRS ≥ 130 ms, cardiac resynchronization therapy (CRT) is indicated per guideline criteria [28]. Independent of LVEF, patients with sustained VT/VF or syncope with documented malignant arrhythmias merit device evaluation; LGE burden, GLS impairment, non-apical extension of trabeculation, and high-risk genotypes (e.g., LMNA) lower the threshold for device implantation when combined with clinical risk [2,3,8,22,23,24]. Management components and triggers for device therapy and anticoagulation are summarized in Table 7.

Anticoagulation is recommended for atrial fibrillation according to thromboembolic risk scores; DOACs are generally preferred, whereas VKA is indicated when apical thrombus is present or DOACs are contraindicated [26,28]. Routine anticoagulation in the absence of AF or thrombus is not supported; decisions should consider recess depth, prior embolism, and global risk. Catheter ablation can reduce recurrent VT when performed in experienced centers using substrate-guided strategies adapted to trabeculated anatomy [3]. Surgical resection of non-compacted myocardium has been reported in highly selected cases with symptomatic improvement and LVEF gain, but evidence remains limited and patient selection is critical [16]. In advanced heart failure, durable LVAD support and cardiac transplantation are established options; outcomes mirror those of other non-ischemic etiologies.

Follow-up intensity is individualized. A reasonable framework is clinical evaluation and imaging every 6 months during the first year after diagnosis or therapy changes, then annually if stable, with earlier reassessment when new symptoms, arrhythmias, or biomarker/imaging changes appear. Exercise: recreational moderate-intensity activity is acceptable in the absence of fibrosis, significant arrhythmias, or systolic dysfunction; participation in high-intensity or competitive sports should follow shared decision-making informed by LVEF, LGE, arrhythmia burden, and genotype [22,23,24,25]. Pregnancy: most women with preserved function and no injury markers tolerate pregnancy, but pre-pregnancy counseling and close surveillance are advisable; postpartum reassessment is recommended when hypertrabeculation appears during gestation [7]. Telemonitoring (rhythm and congestion) can support earlier intervention and reduce hospitalizations in selected patients [29].

## 8. Differential Diagnosis

Several entities can meet echocardiographic or CMR thresholds for LVNC yet represent distinct phenotypes with different prognoses and management. Physiological hypertrabeculation is common in athletes and during late pregnancy and often regresses once the hemodynamic trigger is removed; in these settings, preserved LVEF and absent LGE favor a benign course [7,10]. Apical hypertrophic cardiomyopathy (HCM) can mimic non-compaction due to apical thickening and a spade-like cavity but typically shows a thick compact layer and characteristic LGE distribution. Arrhythmogenic right ventricular cardiomyopathy (ARVC) primarily affects the right ventricle with depolarization/repolarization abnormalities and desmosomal variants; biventricular forms can blur boundaries but differ in electro-anatomic substrate and risk management. Infiltrative/storage diseases such as amyloidosis and Fabry disease produce distinct tissue signatures (e.g., low native T1 and inferolateral mid-wall LGE in Fabry) and systemic features that guide testing and therapy [12,13,15]. Tachycardia-induced cardiomyopathy and other high-output states may cause reversible remodeling; controlling the trigger and reassessing structure/function prevents mislabeling. A multiparametric approach combining morphology with injury markers (LGE, reduced LVEF/GLS) and clinical-genetic context improves specificity and reduces over-diagnosis [2,12,28,30]. Selected differentials and distinguishing clues are presented in Table 8.

## 9. Future Directions and Open Questions

Diagnostic thresholds and risk models for LVNC need prospective validation that accounts for age, sex, ethnicity, and loading conditions. First, criteria standardization should test whether age- and sex-adjusted NC/C or trabeculated-mass cutoffs, when paired with injury markers (LGE, GLS, compact-layer thickness), discriminate pathological from physiological hypertrabeculation across athletes, pregnancy, and high-output states [2,12,28,30]. Recent narrative updates underscore evolving concepts and the need to harmonize diagnostic criteria across populations [31]. Second, genotype-informed risk warrants integration into device and anticoagulation decisions, particularly for high-risk variants such as LMNA, in combination with imaging markers and arrhythmic burden [13,15,22]. Third, advanced imaging and AI-assisted analysis (e.g., 3D CMR segmentation, radiomics, deformation mapping) may improve phenotyping and prediction beyond conventional metrics and should be evaluated in multicenter cohorts with hard outcomes [11,12]. Molecular imaging of fibroblast activation (FAPI) may refine fibrosis phenotyping and risk prediction in cardiomyopathy [32]. Fourth, therapeutic trials should test whether early institution of SGLT2 inhibitors or other cardioprotective agents in asymptomatic genotype-positive individuals can delay fibrosis or adverse remodeling. Fifth, pediatric-to-adult transition research should define progression markers and optimal surveillance intervals, ideally through genotype-enriched registries. Recent reviews highlight evolving concepts and emphasize outcome-anchored, harmonized criteria across physiological states [31]. Finally, global implementation studies must identify simplified imaging protocols and affordable genetic panels that deliver value in resource-limited settings, enabling equitable precision cardiology [29].

## 10. Conclusions

Left ventricular non-compaction cardiomyopathy (LVNC) is best framed as a structural phenotype within a cardiomyopathic continuum rather than a single discrete disease. Echocardiographic and CMR-based morphological thresholds identify the phenotype with reasonable reproducibility, but in low pretest-probability settings such as athletes and late pregnancy the positive predictive value is limited and the risk of overdiagnosis is non-trivial. Diagnostic certainty increases when morphology is paired with injury markers that reflect tissue damage or pump impairment, notably late gadolinium enhancement, reduced ejection fraction or impaired deformation, malignant arrhythmias, or thromboembolism. This multiparametric stance aligns with the underlying biology, where genotype, loading conditions, and mechanobiology interact to shape expression over time.

Clinically, presentation ranges from incidental imaging findings with preserved function to overt heart failure and malignant ventricular arrhythmias. In asymptomatic individuals without injury markers, a conservative strategy that avoids premature labeling and emphasizes periodic surveillance is appropriate. Pregnancy- and training-related hypertrabeculation frequently regresses once the hemodynamic trigger resolves; reassessment 6–12 months postpartum or after a short period of de-training is recommended before assigning a permanent diagnosis. Pediatric LVNC often coexists with congenital or metabolic disease and requires tailored longitudinal plans through the transition to adult care. Across ages, prognosis is driven more by tissue injury and pump function than by trabecular ratios alone; non-apical extension of trabeculation and impaired deformation add granularity for risk stratification even when ejection fraction appears preserved.

Genetics informs both diagnosis and prognosis. Sarcomeric variants connect LVNC with dilated and hypertrophic cardiomyopathies, while nuclear-envelope and conduction-gene variants associate with early conduction disease and ventricular arrhythmias and may lower the threshold for device therapy when combined with imaging or clinical risk features. Genetic testing should be targeted and interpreted within the clinical and imaging context; cascade screening identifies at-risk relatives and clarifies surveillance needs. Advanced imaging and emerging analytics may refine phenotyping and prediction beyond conventional metrics, but require prospective validation with hard outcomes before routine adoption.

Management aligns with non-ischemic cardiomyopathy frameworks. Symptomatic or reduced-ejection-fraction phenotypes merit guideline-directed medical therapy including renin–angiotensin system inhibition or sacubitril–valsartan, beta-blockade, mineralocorticoid receptor antagonists, and SGLT2 inhibitors, titrated to targets as tolerated. After at least three months of optimized therapy, an ejection fraction at or below 35% supports consideration of primary-prevention defibrillator therapy; cardiac resynchronization is indicated in the presence of left bundle branch block and QRS prolongation according to guideline criteria. Independent of ejection fraction, sustained ventricular arrhythmias, extensive or ring-like fibrosis, non-apical extension of trabeculation, high arrhythmic burden, or high-risk genotypes justify earlier device discussion in shared decision-making. Anticoagulation follows standard indications for atrial fibrillation and for documented apical thrombus; catheter ablation can reduce recurrent ventricular tachycardia in selected cases; advanced therapies such as ventricular assist devices and transplantation remain options in end-stage disease, while surgical resection of non-compacted myocardium is exceptional and supported by limited evidence.

Taken together, these data support a standardized, two-step diagnostic framework: first, confirm morphology with attention to technical pitfalls and pretest probability; second, establish disease by demonstrating injury or a plausible high-risk genotype. Care should be personalized by integrating clinical variables, imaging markers, and genotype to calibrate surveillance intensity, device thresholds, anticoagulation, exercise counseling, pregnancy management, and family screening.

## Figures and Tables

**Table 1:** “Apparent prevalence” denotes the proportion of individuals in cohort studies who meet at least one echocardiographic or CMR morphological threshold for LVNC and therefore does not equal disease prevalence. The table was created de novo by the authors from published data; no third-party figures were reproduced. Row-level sources: healthy controls [6]; athletes [10]; pregnancy [7]. Estimates vary with criteria and modality (e.g., echocardiography using Chin or Jenni definitions; CMR using the Petersen diastolic NC/C ≥ 2.3 ratio or Jacquier trabeculated mass > 20%), loading conditions, and segmentation variability; figures should be interpreted as directional signals rather than true population prevalence. In low pretest-probability settings (healthy adults, athletes, pregnancy), morphology alone has limited positive predictive value; a diagnostic label should be considered only when morphology is accompanied by at least one injury marker such as late gadolinium enhancement (LGE), reduced systolic function, malignant arrhythmias, or thromboembolism. Pregnancy- and training-related hypertrabeculation frequently regresses; repeat imaging at 6–12 months postpartum or after a period of de-training is recommended before assigning a permanent diagnosis. The columns summarize, respectively, the screening yield when formal cut-offs are applied, the functional profile typically observed, and the pragmatic clinical action.

Cohort	Apparent Prevalence Meeting ≥ 1 Echocardiographic Criterion	Clinical Note
Healthy controls	~1.05%	Asymptomatic; low pretest probability
Competitive athletes	~3.16%	Preserved LVEF in the vast majority; reassess after de-training if doubt persists
Pregnancy (third trimester)	up to ~18.6%	Predominantly preserved LVEF; regression reported at 1 year postpartum in a subset

Abbreviations: LVEF, left ventricular ejection fraction.

**Table 2:** This table synthesizes the principal genetic categories implicated in LVNC and links them to biology, phenotype, and management. Sarcomeric variants (e.g., MYH7, ACTN2, truncating TTN) support a continuum with DCM/HCM and show variable LVEF and fibrosis across cohorts [2,3,14]. Nuclear-envelope variants, especially LMNA, associate with early conduction disease, atrial/ventricular arrhythmias, and higher arrhythmic risk that may lower thresholds for device therapy [13,15]. Ion-channel/conduction genes (e.g., HCN4, SCN5A) often present with sinus-node dysfunction, PR/QRS prolongation, ventricular arrhythmias, and occasional aortic dilation with HCN4 [5,9,13]. Developmental transcription factors (NKX2-5, PRDM16, TBX20) track with congenital phenotypes and pediatric-onset LVNC [6,13]. Mitochondrial/metabolic defects typically underlie systemic pediatric cardiomyopathies [13]. Injury markers such as LGE and impaired deformation (e.g., reduced GLS) correlate with adverse outcomes and refine risk beyond morphology, complementing genotype-informed risk stratification [1,2,12]. Table created de novo by the authors from published data; no third-party figures were reproduced.

Category	Key Genes (Examples)	Mechanistic Theme	Typical Phenotype(s)	Management Implications
Sarcomeric	MYH7, ACTN2, TTN	Contractile/structural integrity	LVNC overlapping with DCM/HCM; variable LVEF; fibrosis in subsets	HF guideline-directed therapy; family screening; device decisions driven by LVEF/LGE rather than morphology alone
Nuclear envelope	LMNA	Nucleoskeletal signaling; conduction system vulnerability	Early conduction disease; AF/VT; higher arrhythmic burden	Lower threshold for ICD; close rhythm surveillance
Ion channels/conduction	HCN4, SCN5A	Pacemaking and depolarization	Sinus-node dysfunction; PR/QRS prolongation; ventricular arrhythmias; occasional aortic dilation (HCN4)	Ambulatory monitoring; EP referral as needed; aortic imaging if HCN4
Developmental TFs	NKX2-5, PRDM16, TBX20	Cardiac morphogenesis	Association with congenital heart disease; pediatric presentation	Multidisciplinary care; tailored genetic counseling
Mitochondrial/metabolic	mtDNA and nuclear genes	Bioenergetic impairment	Pediatric cardiomyopathy with systemic features	Metabolic work-up; exercise/rehab tailoring

Abbreviations: HF, heart failure; AF, atrial fibrillation; VT, ventricular tachycardia; ICD, implantable cardioverter-defibrillator; LGE, late gadolinium enhancement; LVEF, left ventricular ejection fraction; GLS, global longitudinal strain; EP, electrophysiology; DCM, dilated cardiomyopathy; HCM, hypertrophic cardiomyopathy; QRS, QRS complex duration on surface ECG (ms), PR, atrioventricular conduction interval, TFs, transcription factors; mtDNA, mitochondrial DNA; HCN4, hyperpolarization-activated cyclic nucleotide–gated channel 4 gene.

**Table 3:** This table operationalizes common clinical presentations of LVNC into an initial work-up, pragmatic red flags, and a next action. For incidental CMR findings with preserved LVEF, confirm views/segmentation and re-evaluate morphology with targeted echocardiography (NC/C), strain, and LGE; presence of LGE and reduced GLS indicate tissue injury and track with higher adverse-event rates, while a strong family history supports inherited risk and prompts genetics and closer surveillance [16,21]. For palpitations or syncope, short-term ambulatory monitoring identifies NSVT or sustained VT and conduction disease; high-risk genotypes (e.g., LMNA) and arrhythmia burden justify EP evaluation and, when guideline criteria are met, ICD consideration [3,22,23,24]. For atrial fibrillation or prior embolism, image specifically for apical thrombus and anticoagulate; DOACs are generally preferred, while VKA is indicated when an apical thrombus is present or DOACs are contraindicated [25,26]. For pediatric LVNC, screen for congenital or metabolic disease and tailor follow-up to baseline function and arrhythmias; genetic counseling and cascade screening are recommended to identify at-risk relatives [11,13]. The table prioritizes escalation triggers anchored in injury (LGE, impaired deformation), rhythm/conduction risk, thromboembolism, and family history, aligning clinical action with individualized risk rather than morphology alone. All summary tables were created de novo; no third-party figures were reproduced.

Presentation	Initial Work-Up	Practical Red Flags	Suggested Action
Incidental CMR finding with preserved LVEF	Confirm views/segmentation; targeted echocardiography (NC/C); strain; LGE	LGE present; reduced GLS; strong family history of cardiomyopathy/SCD	Short-interval follow-up; consider electrophysiology and genetics
Palpitations or syncope	24–72 h Holter or patch; echocardiography/CMR	NSVT or sustained VT; conduction disease; high-risk genotype	EP study; ICD consideration if additional criteria are met
Atrial fibrillation or prior embolism	TTE/CMR focused on apical thrombus; thromboembolic risk scores	Apical thrombus; recurrent embolism; low LVEF	Anticoagulation (DOAC preferred; VKA if apical thrombus or contraindications)
Pediatric LVNC phenotype	Echo/CMR; genetic panel; screening for syndromic features	Congenital defects; progressive systolic dysfunction; ventricular arrhythmias	Multidisciplinary pediatric cardiomyopathy care; family screening

Abbreviations: LVEF, left ventricular ejection fraction; LGE, late gadolinium enhancement; GLS, global longitudinal strain; EP, electrophysiology; SCD, sudden cardiac death; TTE, transthoracic echocardiography; DOAC, direct oral anticoagulant; VKA, vitamin K antagonist; NSVT, non-sustained ventricular tachycardia; ICD, implantable cardioverter-defibrillator; NC/C, non-compacted/compacted ratio; CMR, cardiac magnetic resonance; Echo, echocardiography (transthoracic unless specified); VT, ventricular tachycardia.

**Table 4:** This table summarizes the most commonly used morphological criteria for LVNC and their practical pitfalls across imaging modalities. Echocardiographic thresholds (Chin end-diastolic ratio; Jenni systolic NC/C with a bilayered myocardium and perfused recesses) are simple and widely taught, but their specificity drops in low pretest-probability settings and under load-dependent conditions, and they are sensitive to apical foreshortening, caliper placement, and through-plane motion [10,15]. CMR improves reproducibility and whole-heart coverage; the diastolic Petersen NC/C ≥ 2.3 and the Jacquier trabeculated-mass fraction > 20% have been validated in cohorts, yet reader experience, segmentation choices, and vendor/software differences introduce variability and can shift classification rates [7,8]. Because morphology alone has limited positive predictive value in many real-world scenarios, decision-making should be anchored in injury markers—fibrosis on LGE, reduced LVEF/GLS, or malignant arrhythmias/thromboembolism—which consistently track with adverse outcomes and refine risk beyond structural ratios [1]. A pragmatic approach is to confirm morphology across modalities when feasible, incorporate tissue and functional injury signals, and interpret results in light of clinical context and genotype before assigning a durable diagnostic label. All summary tables were created de novo; no third-party figures were reproduced.

Method	Core Definition	Strengths	Caveats/False Positives	When to Use
Chin (echo)	Apical ratio X/Y ≤ 0.5 in diastole	Simple; widely taught	Load-dependent; variable reproducibility; apical foreshortening	Initial screening; confirm with CMR if doubt
Jenni (echo)	NC/C > 2 in systole; bilayered myocardium; perfused recesses	Adds physiological detail	Specificity drops in athletes and pregnancy	Moderate/high pretest settings with adequate windows
Petersen (CMR)	Diastolic NC/C ≥ 2.3	Reproducible; whole-heart coverage	May overcall if used alone; reader/segmentation effects	Baseline CMR characterization
Jacquier (CMR)	Trabeculated mass > 20% LV mass	Quantifies burden	Segmentation and software variability; thresholds cohort-dependent	Follow-up quantification; research and complex cases
Risk modifiers	LGE; reduced GLS; thin compact layer	Improve risk classification	Not diagnostic alone	Combine with morphology to finalize label

Abbreviations: NC/C, non-compacted/compacted ratio; LVEF, left ventricular ejection fraction; LGE, late gadolinium enhancement; GLS, global longitudinal strain; CMR, cardiac magnetic resonance; LV, left ventricle, X/Y (Chin ratio): on echocardiography, X is the distance from the epicardial surface to the trough of the deepest inter-trabecular recess; Y is the distance from the epicardial surface to the peak of the trabeculation. Assessed at end-diastole in short-axis, LVNC if X/Y ≤ 0.5.

**Table 5:** This table outlines contexts in which physiological hypertrabeculation is common and LVNC thresholds can be met without true disease. In endurance/strength athletes, increased trabeculation with preserved LVEF and absent LGE typically represents adaptive remodeling; short-term de-training followed by reassessment helps avoid over-diagnosis [10]. In late pregnancy, hemodynamic loading may transiently satisfy morphological cut-offs, with postpartum regression frequently observed; repeat imaging at 6–12 months is advised before assigning a permanent label [7]. Hyperdynamic states (e.g., anemia, thyrotoxicosis) can similarly accentuate trabeculation and should be re-evaluated after the trigger is treated. During adolescence/early adulthood, transitional remodeling may blur thresholds, so multiparametric assessment (strain, LGE) is recommended. Across these settings, morphology by itself has low positive predictive value; defer diagnosis until injury markers or persistent remodeling are demonstrated, integrating family/genetic data where relevant [2,7,10,12,20]. All summary tables were created de novo; no third-party figures were reproduced.

Context	Typical Features	Suggested Follow-Up
Endurance/strength athletes	Increased trabeculation; preserved LVEF; absent LGE	Reassess after a period of de-training if uncertainty remains
Pregnancy (third trimester)	Increased wall stress; variable trabeculation	Reassess 6–12 months postpartum before assigning a permanent diagnosis
Hyperdynamic states (e.g., anemia)	High cardiac output; reversible remodeling	Treat trigger; reassess morphology after stabilization
Adolescence/early adulthood	Transitional remodeling	Multiparametric assessment (strain, LGE)

Abbreviations: LVEF, left ventricular ejection fraction; LGE, late gadolinium enhancement.

**Table 6:** This table integrates imaging, electrical, and genetic markers that modify prognosis in LVNC. LGE is a reproducible tissue marker linked to ≈2-fold higher adverse events independently of LVEF [2,8]. LVEF ≤ 35% after optimized guideline-directed therapy remains the cornerstone threshold for primary-prevention ICD in non-ischemic cardiomyopathy [28], while extensive/ring-like LGE and non-apical extension of trabeculation signal higher risk and justify intensified rhythm surveillance and earlier device consideration [2,8,22]. Genotype can up- or down-shift risk; LMNA variants exemplify conduction disease and arrhythmic vulnerability that lower the ICD threshold when combined with imaging or clinical risk features [13,15,22]. Reduced GLS often precedes overt LVEF decline and, together with LGE, refines risk in “preserved-EF” phenotypes [12]. Thromboembolic risk rises with AF and apical thrombus and mandates anticoagulation, preferably with a DOAC unless a visible thrombus or contraindication dictates VKA [22,25]. Table created de novo by the authors from published data; no third-party figures were reproduced.

Marker	Evidence/Association	Clinical Implications
LGE present	≈2-fold higher risk of adverse events across LVNC cohorts, independent of LVEF [2,8]	Closer follow-up; consider ICD when combined with low LVEF, high arrhythmic burden, or high-risk genotype
LVEF ≤ 35% after optimized GDMT	Standard high-risk threshold in non-ischemic cardiomyopathy [28]	Primary-prevention ICD per guidelines; optimize GDMT and consider CRT if criteria are met
Extensive or ring-like LGE	Higher arrhythmic/MACE risk in cardiomyopathy populations, signal reproduced in LVNC series [2,8]	Prioritize device therapy and rhythm surveillance; cautious approach to high-intensity sports
Non-apical extension of trabeculation	Tracks with adverse remodeling and events in observational LVNC cohorts [22]	Tighter surveillance; integrate with LGE/LVEF/GLS and genotype to guide ICD decisions
High-risk genotype (e.g., LMNA)	Early conduction disease and ventricular arrhythmias; higher SCD risk [13,15,22]	Lower threshold for ICD; ambulatory rhythm monitoring; family screening
Reduced GLS (impaired deformation)	Identifies subclinical dysfunction and correlates with fibrosis/bad outcomes [12]	Escalate follow-up and GDMT even if LVEF is “preserved”; consider CMR if not already done
Atrial fibrillation/apical thrombus	Increased thromboembolic risk in LVNC cohorts [22]	Anticoagulation (DOAC preferred; VKA if apical thrombus or DOAC contraindication)

Abbreviations: LGE, late gadolinium enhancement; LVEF, left ventricular ejection fraction; GLS, global longitudinal strain; ICD, implantable cardioverter-defibrillator; CRT, cardiac resynchronization therapy; GDMT, guideline-directed medical therapy; AF, atrial fibrillation; DOAC, direct oral anticoagulant; VKA, vitamin K antagonist; MACE, major adverse cardiovascular events; SCD, sudden cardiac death.

**Table 7:** This table consolidates therapeutic decisions for LVNC by aligning heart-failure frameworks with LVNC-specific risk modifiers. GDMT remains foundational for symptomatic patients and those with reduced LVEF [28]. ICD and CRT indications follow non-ischemic cardiomyopathy guidance, with thresholds individualized by LGE, GLS, non-apical extension, and genotype (e.g., LMNA) when risk is borderline [2,3,8,22,23,24,25]. Anticoagulation is mandatory for AF and for apical thrombus (prefer VKA when thrombus is present); DOACs are preferred otherwise [26,28]. Catheter ablation reduces VT recurrence in selected LVNC patients [3]; LVAD and transplantation are options in advanced disease, while surgical resection of non-compacted myocardium is reserved for exceptional, highly selected cases with limited evidence [16]. Table created de novo by the authors from published data; no third-party figures were reproduced.

Clinical Situation	Recommended Strategy	Practical Notes/Triggers
Asymptomatic, preserved LVEF, no injury markers	Periodic follow-up: annual echo or CMR + ambulatory monitoring	Defer label/device; escalate only if LGE/GLS abnormality, arrhythmias, or remodeling appear [2,12]
Symptomatic HFrEF or LVEF ↓	GDMT: ACEi/ARB or ARNI + beta-blocker + MRA + SGLT2i	Titrate to guideline doses as tolerated; manage congestion; cardiac rehab improves capacity [28]
LVEF ≤ 35% despite ≥3 months GDMT	ICD for primary prevention	Consider CRT if LBBB with QRS ≥ 130 ms; integrate LGE/GLS/genotype for borderline cases [28]
Sustained VT/VF or syncope with malignant arrhythmias	ICD ± catheter ablation	Substrate-guided ablation can reduce VT recurrence; experienced centers recommended [3,23,24]
Atrial fibrillation or prior embolism	Anticoagulation (DOAC preferred)	VKA if apical thrombus present or DOAC contraindicated; image to confirm thrombus [26,28]
High-risk genotype (e.g., LMNA) with additional risk features	Lower threshold for device therapy	Combine genotype with LGE/GLS, conduction disease, family history to personalize ICD decision [13,15,22]
Advanced heart failure	LVAD or transplantation	Outcomes comparable to other NICM etiologies; rare surgical resection in selected LVNC cases [16]
Return to sport / lifestyle	Shared decision-making based on LVEF, LGE, arrhythmias, genotype	Recreational moderate exercise acceptable if no injury markers; restrict competitive sports if high risk [22,23,24,25]

Symbols: The downward arrow (↓) denotes a decrease/reduced value of the parameter it follows. Thus, “LVEF ↓” = reduced left ventricular ejection fraction (i.e., below normal or at the threshold specified in the cell, e.g., ≤35% where indicated). Abbreviations: GDMT, guideline-directed medical therapy; ACEi, angiotensin-converting enzyme inhibitor; ARB, angiotensin receptor blocker; ARNI, angiotensin receptor–neprilysin inhibitor; MRA, mineralocorticoid receptor antagonist; SGLT2i, sodium-glucose cotransporter-2 inhibitor; LVEF, left ventricular ejection fraction; LGE, late gadolinium enhancement; GLS, global longitudinal strain; ICD, implantable cardioverter-defibrillator; CRT, cardiac resynchronization therapy; VT, ventricular tachycardia; VF, ventricular fibrillation; DOAC, direct oral anticoagulant; VKA, vitamin K antagonist; LVAD, left ventricular assist device; NICM, non-ischemic cardiomyopathy; LBBB, left bundle branch block. HFrEF, heart failure with reduced ejection fraction; QRS, QRS complex duration on surface ECG (ms).

**Table 8:** This table outlines conditions that can satisfy LVNC thresholds yet have different pathophysiology and management. In athletes and pregnancy, hypertrabeculation is frequently reversible and rarely accompanied by injury markers (LGE, reduced LVEF/GLS) [7,10]. Apical HCM shows a thick compact layer and a characteristic CMR fibrosis pattern; ARVC presents a right-dominant arrhythmogenic substrate; Fabry shows low native T1 and systemic features; and amyloidosis has diffuse infiltration on CMR. When morphology is borderline, pairing injury markers with genotype/clinical context improves diagnostic specificity and helps avoid overdiagnosis [2,12,28,30]. Table created de novo by the authors; no third-party figures were reproduced.

Entity	Distinguishing Clues	Tests That Help	What Rules LVNC In/Out
Physiological hypertrabeculation (athletes, pregnancy)	Preserved LVEF; no LGE; reversible after trigger	De-training or postpartum reassessment	Regression and absence of injury markers argue against LVNC [2,7,10]
Apical HCM	Apical hypertrophy; spade-like cavity	CMR wall thickness; characteristic LGE	Thick compact layer favors HCM over LVNC; pattern of fibrosis differs
Fabry disease	Neuropathic pain; cornea verticillata; proteinuria	Low native T1; GLA testing	Storage-disease pattern and inferolateral LGE point away from LVNC [12,15]
ARVC	RV-dominant phenotype; epsilon waves; desmosomal variants	RV-focused CMR criteria; genetic testing	Predominant RV substrate distinguishes from classic LVNC
Cardiac amyloidosis	Low-voltage ECG; diffuse subendocardial LGE	Bone-avid tracers; biopsy as indicated	Diffuse infiltration rather than trabecular morphology
Tachycardia-induced cardiomyopathy	Persistent tachyarrhythmia; reversibility	Rhythm control; remodeling on follow-up	Structural/functional recovery argues against LVNC

Abbreviations (Section 8): LVEF, left ventricular ejection fraction; LGE, late gadolinium enhancement; GLS, global longitudinal strain; HCM, hypertrophic cardiomyopathy; ARVC, arrhythmogenic right ventricular cardiomyopathy; CMR, cardiac magnetic resonance; RV, right ventricle, T1, native myocardial T1 mapping (non-contrast CMR); GLA, alpha-galactosidase A (enzyme activity) and/or GLA gene testing (Fabry disease), ECG, electrocardiogram.

## Data Availability

No new data were created or analyzed in this study. Data sharing is not applicable to this article.

## Abbreviations

The following abbreviations are used in this manuscript:

LVNC	Left-ventricular non-compaction cardiomyopathy
CMR	Cardiac magnetic resonance
LGE	Late gadolinium enhancement
LVEF	Left-ventricular ejection fraction
ICD	Implantable cardioverter-defibrillator
CRT	Cardiac resynchronization therapy
SGLT2i	Sodium–glucose co-transporter-2 inhibitor
DCM	Dilated cardiomyopathy
HCM	Hypertrophic cardiomyopathy
NC/C	Non-compacted/compacted ratio
NT-proBNP	N-terminal pro-B-type natriuretic peptide
